# MOG-Ab-associated optic neuritis with concurrent GFAP-Ab positivity following tislelizumab-based treatment: a case report

**DOI:** 10.3389/fphar.2026.1834795

**Published:** 2026-07-02

**Authors:** Yinghui Zhang, Yonghu Liu, Hongxia Xin, Qin Shi, Qiang Liu, Wenna Zhao, Zhuoxiu Qi, Gang Ma, Xiao Yang

**Affiliations:** 1 Department of Neurology, General Hospital of Ningxia Medical University, Yinchuan, Ningxia, China; 2 Department of Respiratory and Critical Care Medicine, General Hospital of Ningxia Medical University, Yinchuan, Ningxia, China; 3 Department of Ophthalmology, General Hospital of Ningxia Medical University, Yinchuan, Ningxia, China; 4 The First Clinical Medical College, Ningxia Medical University, Yinchuan, Ningxia, China

**Keywords:** extensive-stage small cell lung cancer, GFAP-Ab, immune-related adverse events, MOG-Ab-associated ON, PD-1 inhibitor, tislelizumab

## Abstract

**Objective:**

To investigate the clinical characteristics and therapeutic management of myelin oligodendrocyte glycoprotein antibody (MOG-Ab)-related optic neuritis (ON), a rare neurological immune-related adverse event (irAE) associated with the programmed death-1 (PD-1) inhibitor tislelizumab.

**Methods:**

We report a retrospective case of a 48-year-old male patient with extensive-stage small cell lung cancer (ES-SCLC) who developed acute bilateral vision loss after receiving the first dose of tislelizumab combined with chemotherapy. After admission to the neurology department and a systematic clinical evaluation, the patient was found to be positive for both MOG-Ab and glial fibrillary acidic protein antibody (GFAP-Ab). He was ultimately diagnosed with MOG-Ab-associated ON with Concurrent GFAP-Ab Positivity.

**Results:**

Following diagnosis, tislelizumab was promptly discontinued. The patient received high-dose methylprednisolone (1,000 mg/d) as initial therapy, followed by sequential tapering combined with rituximab for immunomodulation. This regimen resulted in considerable visual recovery. In subsequent treatment lines, immune checkpoint inhibitors (ICIs) were avoided.

**Conclusion:**

This case underscores the potential of PD-1 inhibitors to trigger rare MOG-Ab-associated ON. For such vision-threatening irAEs, early recognition, immediate ICI withdrawal, multidisciplinary collaboration, and aggressive immunosuppressive therapy are essential for reversing neurological impairment and improving outcomes.

## 1 Introduction

Lung cancer remains the most prevalent and lethal malignancy globally, imposing a substantial disease burden (Sung et al., 2021), with its most aggressive histological subtype, Small cell lung cancer (SCLC), accounting for approximately 13%–15% of all lung cancer cases and classified according to disease extent as limited-stage SCLC (LS-SCLC) or extensive-stage SCLC (ES-SCLC), the latter representing the majority of initial presentations and bearing an exceedingly poor prognosis (Gazdar et al., 2017; Zhang et al., 2023). The combination of chemotherapy with immune checkpoint inhibitors (ICIs), represented by tislelizumab—an inhibitor of programmed death-1 (PD-1)—has become the standard first-line treatment for ES-SCLC, offering a marked survival benefit (Yi et al., 2024). Nevertheless, through immune activation, ICIs, including those targeting cytotoxic T-lymphocyte-associated protein 4, PD-1, and programmed cell death ligand 1, may also induce immune-related adverse events (irAEs) (Haanen et al., 2022). While irAEs frequently affect the skin, gastrointestinal tract, and endocrine system, neurological manifestations, such as optic neuritis (ON), are relatively uncommon (Blum et al., 2023; Puzanov et al., 2017; Ramos-Casals et al., 2020; Carr et al., 2025). Myelin oligodendrocyte glycoprotein antibody-associated disease (MOGAD) is an inflammatory demyelinating disorder of the central nervous system (CNS) mediated by immunoglobulin G (IgG) antibodies targeting myelin oligodendrocyte glycoprotein that predominantly affects children and young adults (Gutierrez et al., 2025; Qiu et al., 2020). Although a recent review (Andersen and Brilot, 2025) systematically summarized its potential pathogenesis, a definitive expert consensus remains to be established. The disease exhibits considerable phenotypic heterogeneity—including ON, myelitis, and encephalitis—which can lead to initial diagnostic challenges (Qiu et al., 2020). Notably, the association between PD-1 inhibitors and MOGAD remains clinically underrecognized owing to the paucity of reported cases. Here, we describe a rare case of severe MOGAD occurring within 24 h of the initial administration of tislelizumab and chemotherapy in a middle-aged male patient with ES-SCLC. This report contributes to the limited literature regarding this severe irAE and provides practical insights for the identification and management of such critical complications during ICI therapy. This analysis aims to enhance awareness of cancer therapy-induced MOGAD, thereby facilitating informed clinical decision-making.

## 2 Case description

A 48-year-old man presented with a 1-week history of chest pain and dyspnea, which progressed to include fever, fatigue, and limb weakness. He was admitted to the Department of Respiratory and Critical Care Medicine on 27 August 2025. Contrast-enhanced chest CT revealed a soft tissue mass obstructing the left lower lobe bronchus, with associated distal consolidation, mediastinal lymphadenopathy, and left pleural effusion/thickening suggestive of metastasis (Figures 1A,B). Bronchoscopy identified an obstructive tumor (Figure 1C). Histopathological (Figure 1D) and immunohistochemical analysis of the biopsy confirmed central-type SCLC. Staging confirmed pleural involvement (cT3N2M1) without distant metastasis, leading to a diagnosis of ES-SCLC.

**FIGURE 1:**
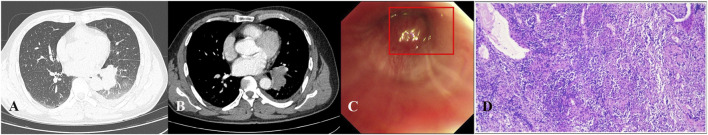
Contrast-enhanced chest computed tomography (CT) and bronchoscopic findings. **(A,B)** Contrast-enhanced chest CT (26 August 2025) demonstrating a soft-tissue mass in the anteromedial basal segment of the left lower lobe with surrounding infiltrative opacities and a small left pleural effusion **(A)**, lung window; **(B)**, mediastinal window). **(C,D)** Bronchoscopy and biopsy findings (28 August 2025). **(C)** Bronchoscopic view showing an infiltrative neoplasm occluding the lumen of the lateral basal segment in the left lower lobe. **(D)** Frozen-section histopathology (hematoxylin and eosin staining, ×100) of the bronchial biopsy specimen, revealing mucosal tissue with areas of crushed cells.

The patient subsequently received chemotherapy combined with immunotherapy. On 3 September 2025, he received the first treatment cycle, comprising etoposide (150 mg, days 1–3), carboplatin (AUC 4, 800 mg, day 1), and tislelizumab (200 mg, day 1). The next day (September 4), the patient developed acute bilateral blurred vision accompanied by periorbital pain, prompting an urgent ophthalmological consultation. Specialized examination revealed corrected visual acuity of 0.4 in the right eye and 0.1 in the left eye. Bilateral intraocular pressure was 8 mmHg. Mild bilateral eyelid edema was noted, while conjunctival injection or corneal abnormalities were absent. Pupillary examination revealed equal, round pupils (∼3 mm) that were reactive to light, with clear lenses bilaterally. Fundus photography showed optic discs with clear margins and normal coloration, with a cup-to-disc (C/D) ratio of approximately 0.3; although retinal vessels appeared normal, extensive punctate hemorrhages were observed in the macula. Macular optical coherence tomography (OCT) confirmed the presence of bilateral cystoid macular edema (Figures 2A–C). The patient was diagnosed with bilateral retinal vein occlusion (RVO) and macular edema. He was advised to continue aggressive anti-tumor therapy, scheduled for outpatient follow-up, and discharged on 8 September 2025.

**FIGURE 2:**
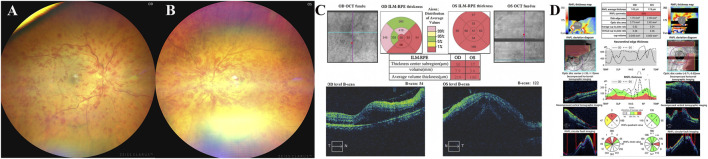
Fundus and optical coherence tomography (OCT) findings. **(A,B)** Fundus photographs of the right (Oculus dexter, OD) and left (Oculus sinister, OS) eyes on 4 September 2025, showing optic discs with clear margins, a pale red color, and a C/D ratio of approximately 0.3. Retinal vessels show a normal course, with extensive punctate hemorrhages involving the macula. **(C)** Macular OCT of both eyes on the same date revealed cystoid macular edema. **(D)** Optic nerve head OCT of both eyes on 24 September 2025, showing thickening of the retinal nerve fiber layer (RNFL) and severe edema of both optic discs.

On 22 September 2025, the patient returned to the ophthalmology clinic due to rapidly worsening visual acuity, reporting subjective No Light Perception in both eyes. Examination revealed uncorrected visual acuity of counting fingers at one foot in the right eye and hand motion in the left eye. Intraocular pressure was 10 mmHg bilaterally. The corneas and anterior chambers were clear. Both pupils were equally dilated and round (5 mm) with sluggish light reflexes. Mild lens opacity and diffuse fine vitreous opacities were noted. Fundus photography demonstrated severe bilateral papilledema, characterized by hyperemic discs with blurred margins, numerous retinal hemorrhages, yellow-white exudates, and vascular tortuosity. Bilateral macular OCT showed heterogeneous reflectivity, while optic nerve head OCT confirmed marked thickening of the peripapillary retinal nerve fiber layer (RNFL) and marked papilledema (Figure 2D). Bilateral ocular ultrasound revealed vitreous opacities and abnormal optic nerve heads; consequently, the diagnosis was revised to optic papillophlebitis. Given the rapid progression and poor response to conventional treatment, the patient was referred to the neurology clinic for further comprehensive evaluation. Head and neck computed tomography angiography revealed no substantial abnormalities. Lumbar puncture results were as follows: opening pressure, 150 mmH_2_O; routine cerebrospinal fluid (CSF) analysis: Pandy test positive; total cells, 450 cells/mm^3^; white blood cell count, 80 cells/mm^3^. CSF cytology: clear and colorless; white blood cell count, 92 cells/mm^3^ (lymphocytes 84%, monocytes 10%, neutrophils 2%, plasma cells 4%). CSF IgG was 87.8 mg/L. Biochemical examination indicated an elevated CSF protein level of 1.28 g/L (reference range: 0.12–0.60 g/L).

The patient was admitted to the neurology department on 25 September 2025, for evaluation of unexplained visual loss. Neurological examination demonstrated bilaterally diminished extraocular movements in all directions and absent direct, consensual, and accommodation pupillary reflexes. No other focal neurological deficits were found. Visual evoked potentials showed abnormalities bilaterally. Brain magnetic resonance imaging (MRI) with contrast and diffusion-weighted imaging (DWI) revealed no notable abnormalities. Orbital MRI with contrast and DWI demonstrated bilateral optic nerve thickening as evidenced by increased DWI signal intensity, accompanied by mild enhancement of the left optic nerve sheath and enhancement of the right optic nerve sheath (Figures 3A–D). On 27 September 2025, testing for demyelinating antibodies via cell-based assay revealed the following titers: serum MOG-Ab 1:32 and glial fibrillary acidic protein antibody (GFAP-Ab) 1:10; CSF MOG-Ab 1:1 and GFAP-Ab 1:3.2 (See supplementary material). Additionally, further testing of CSF and serum for Oligoclonal Bands (OBs) showed both were type 4. Given the clear temporal association with tislelizumab infusion, acute bilateral visual loss, characteristic ophthalmologic imaging findings (bilateral optic nerve thickening, RNFL thickening, papilledema, macular edema), and positivity for MOG-Ab in both serum and CSF, a final diagnosis of MOGAD-related ON occurring in the setting of tislelizumab-based treatment, with concurrent GFAP-Ab positivity, was established. Importantly, the patient did not exhibit typical anti-GFAP-Ab-associated clinical and radiological features, and the GFAP-Ab titers in serum and CSF did not reach the diagnostic threshold for clinical significance. Tislelizumab was permanently discontinued. Management included intravenous methylprednisolone pulse therapy (1,000 mg daily) followed by a tapering regimen. To prevent relapse, rituximab (500 mg) was administered intravenously on 9 October 2025. Following treatment, the patient’s condition stabilized with measurable improvement in visual acuity compared to baseline. He was discharged on a tapering course of oral prednisone (60 mg daily). No marked discomfort was reported during follow-up. Subsequent antitumor therapy was continued with platinum-based chemotherapy plus etoposide, while tislelizumab was not reintroduced.

**FIGURE 3:**
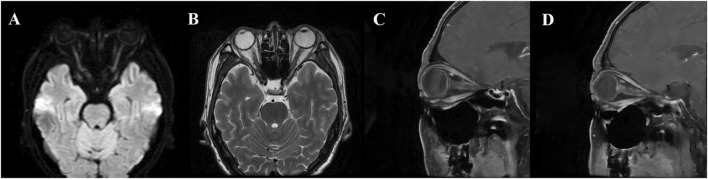
Orbital contrast-enhanced magnetic resonance imaging (MRI) + diffusion-weighted imaging (DWI) on 27 September 2025. **(A)** The DWI signal is increased in both optic nerves. **(B)** T2WI showing bilateral optic nerve thickening with slightly increased signal in the left optic nerve. **(C)** T1+C showing mild enhancement of the left optic nerve sheath. **(D)** T1+C showing enhancement of the right optic nerve sheath.

On 11 October 2025, the patient received a second cycle of chemotherapy with the carboplatin (AUC4, 700 mg on day 1) plus etoposide (150 mg on days 1–3) regimen, which was tolerated well. The patient continued with regular subsequent chemotherapy. On 7 November 2025, during hospitalization for the third cycle, a follow-up contrast-enhanced chest CT showed that the soft-tissue mass in the lateral basal segment of the left lower lobe had decreased in size. The patient was re-evaluated in the ophthalmology clinic on 26 November 2025. Uncorrected visual acuity was 0.4 in the right eye and 0.3 in the left eye, with normal bilateral intraocular pressure. Fundus examination revealed bilateral optic discs with gradually clearer margins, a pale red color, no obvious hemorrhages or exudates were observed, and the macular light reflex was present. Repeat macular OCT of both eyes showed no obvious abnormalities. Optic nerve head OCT demonstrated slightly deepened optic cups, with C/D ratios of 0.60 for the right eye and 0.66 for the left eye. The patient’s condition was assessed as improved and stable.

## 3 Discussion and literature review

Early studies suggested that the incidence of ocular immune-related adverse events (OirAEs) associated with ICIs was below 1%. Subsequent data have suggested an actual incidence ranging from 2.8% to 4.3%, with clinical manifestations including dry eye, uveitis, ON, and keratitis. Among these, optic neuropathy accounts for approximately 8.4% of all OirAEs, with ON induced by PD-1 inhibitors being particularly rare (Puzanov et al., 2017; Wladis and Kambam, 2022). To date, fewer than five cases of severe intraocular inflammation related to tislelizumab, a PD-1 inhibitor developed in China, have been reported (Li et al., 2024). Although MOGAD frequently presents as ON, tislelizumab-induced MOGAD has not been previously documented in the literature. This case report describes the first instance of this novel irAE and its favorable therapeutic outcome, providing valuable insights for the clinical management of such serious complications.

This case establishes a link between the PD-1 inhibitor tislelizumab and the induction of MOGAD, an exceptionally rare complication. The patient developed acute bilateral vision loss within 24 h of the initial tislelizumab cycle for ES-SCLC, which progressively worsened over the following 19 days to complete loss of light perception, indicating that extensive immune activation had occurred. Clinical findings included bilateral retinal hemorrhages, macular and optic disc edema, and RNFL thickening, which were highly suggestive of an irAE. Orbital MRI confirmed bilateral optic nerve thickening with diffusion restriction and sheath enhancement, thus localizing the pathology. Critically, serum and CSF analyses revealed that MOG antibodies were elevated (serum titer 1:32), findings that are consistent with MOG-Ab-associated ON. The close temporal link to drug administration and the significant clinical improvement observed upon tislelizumab discontinuation and immunosuppressive therapy indicate that a causal relationship exists between tislelizumab and this rare autoimmune response. These findings suggest that ICIs may breach immune tolerance, resulting in autoimmunity against neural antigens such as MOG. How are pathogenic MOG-Ab produced?

The pathogenic MOG-Ab may not necessarily arise within 24 h after drug administration; rather, a pre-existing subclinical autoimmune response may have been acutely activated. For example, Stephanie et al. (Syc-Mazurek et al., 2024) reported a case of a middle-aged male with melanoma and progressive encephalomyelitis who already had serum MOG-Ab at a titer of 1:1,000 and CSF MOG-Ab at 1:4,096. After treatment, the patient developed metastases and subsequently experienced ON and myelitis following tislelizumab therapy. This case illustrates that the potent immune-activating effects of ICIs can disrupt immune homeostasis, thereby triggering or unmasking latent autoimmune disorders. Moreover, the onset of MOGAD is associated with the activation of MOG-Ab. In susceptible individuals, MOG-specific autoreactive T cells and B cells may already exist in a quiescent or subclinical state. ICIs break immune tolerance by releasing inhibitory signals on T cells, which can lead to rapid and robust activation and expansion of these pre-existing autoreactive lymphocytes. This process does not require a complete *de novo* adaptive immune response and can occur within hours to days (Syc-Mazurek et al., 2024; Corbali and Chitnis, 2023). Other strong immune stimuli, such as Corona Virus Disease 2019 infection, have also been reported to trigger acute MOGAD within a short period (Assavapongpaiboon et al., 2022). Thus, the rapid bilateral vision loss in this patient likely resulted from tislelizumab rapidly amplifying a pre-existing, low-level autoimmune response against MOG (Syc-Mazurek et al., 2024; Corbali and Chitnis, 2023).

Consistent with acute immune-mediated ON, the observed retinal vascular changes can be explained by secondary compromise due to direct inflammatory extension from the optic nerve. MOG-Ab binds to MOG on oligodendrocytes and myelin, activating complement and membrane attack complexes, which cause perivascular inflammation and demyelination (Misu, 2025). This inflammation can extend to vessel walls, leading to retinal vasculitis, increased permeability, and hemorrhage. Severe optic disc edema, a hallmark of MOGAD-related ON (Dolan et al., 2026), may directly extend to peripapillary retinal tissues and vessels, inducing venous stasis, tortuosity, exudation, and hemorrhages. Finally, synergistic T-cell and B-cell responses amplify tissue inflammation, allowing the process to spread from the optic nerve to adjacent retina (Lerch et al., 2023). Thus, these vascular changes are secondary inflammatory phenomena arising from direct extension of the underlying MOG-Ab-associated ON, rather than a separate entity (Saify et al., 2025).

A prominent feature of this case was the dual positivity of MOG-Ab and GFAP-Ab in both serum and CSF, with serum titers of 1:32 for MOG-Ab and 1:10 for GFAP-Ab, and CSF titers of 1:1 for MOG-Ab and 1:3.2 for GFAP-Ab. The overlap syndrome involving these two antibodies is relatively rare compared with overlaps involving other antibodies such as aquaporin-4 antibody or N-methyl-D-aspartate receptor antibody (Flanagan et al., 2017; Shan et al., 2018; Zhong et al., 2025). A 2024 review by Fang et al. indicated that the primary clinical manifestation of MOG-Ab and GFAP-Ab co-positivity is meningoencephalomyelitis (62.5%), with fever, bladder dysfunction, and headache being the most prevalent symptoms (62.5% each), followed by visual decline (50%). Imaging studies revealed features of both diseases in affected patients (Li and Chen, 2025; Fang et al., 2024). Mechanistically, MOG-Ab commonly involves the optic nerve, whereas autoimmune GFAP astrocytopathy (A-GFAP-A) is typically associated with prominent meningeal involvement. Furthermore, the diagnosis of A-GFAP-A relies heavily on CSF testing, as CSF GFAP-Ab exhibits higher disease specificity than low-titer serum GFAP-Ab (Zhang et al., 2025; Hagbohm et al., 2024; Jiajia et al., 2024; Chinese Medical Association Neurology Branch Neuroimmunolo gy Group, 2023). Although a unified quantitative diagnostic threshold for A-GFAP-A has not yet been established, a 2025 study demonstrated that a CSF GFAP-IgG antibody titer grade ≥4 (typically corresponding to a titer ≥1:10) is an independent risk factor for distinguishing A-GFAP-A from other GFAP-IgG-positive encephalitis (Zhang et al., 2025). In the present case, however, the patient presented clinically with isolated acute ON and no clinical or imaging evidence of meningoencephalitis or myelitis. Moreover, the patient’s CSF GFAP-Ab titer (1:3.2) was below the aforementioned risk threshold. Additional studies have shown that when low-titer GFAP-Ab coexists with another well-established pathogenic antibody (e.g., aquaporin-4 antibody or N-methyl-D-aspartate receptor antibody), the clinical phenotype is more likely driven by the latter (Kim et al., 2023; Liu and Zhang, 2025). Taken together, these findings suggest that in this case, MOG-Ab was the primary pathogenic antibody, whereas the presence of GFAP-Ab may lack independent pathogenic significance. The coexistence of both antibodies in this patient likely reflects that ICI-triggered immune attacks simultaneously target distinct glial components, a finding that contributes to our understanding of the complexity of ICI-associated neuroimmune reactions (Cirkel et al., 2021).

This case underscores the diagnostic challenges posed by OirAEs, which can closely mimic common conditions such as RVO. The initial diagnostic misdirection emphasizes the importance of considering irAEs in patients presenting with atypical ocular manifestations during ICI therapy (Ip and Hendrick, 2018). However, establishing an irAE diagnosis is only the initial step; accurate phenotypic classification is essential, as management strategies vary considerably across subtypes. This distinction is exemplified by comparing our case with the recently reported Vogt-Koyanagi-Harada-like syndrome (VKHLS) induced by ICIs (Ding et al., 2025). Although both conditions present with bilateral visual impairment, they differ fundamentally in immunopathogenesis and clinical features. While MOGAD is an antibody-mediated disorder directed against CNS myelin, VKHLS is primarily T-cell mediated and targets uveal melanocytes. Clinically, the patient presented mainly with acute optic nerve dysfunction, in contrast to the prominent anterior uveitis characteristic of VKHLS. Ultimately, positivity for serum MOG antibodies provided definitive laboratory evidence confirming MOG-ON and excluding VKHLS or other mimics (Al-Ani et al., 2023).

Furthermore, contrast-enhanced brain MRI performed during staging of the patient’s SCLC showed no intracranial metastatic lesions, thereby excluding CNS tumor metastasis. The imaging also revealed no characteristic multiple sclerosis (MS) lesions in periventricular, cortical, or brainstem regions, and the patient did not meet criteria for dissemination in space or time. The presence of type 4 OBs in both serum and CSF provided further evidence against MS (Moccia et al., 2025). SCLC is frequently associated with paraneoplastic neurologic syndromes (PNS), and although the patient’s acute bilateral visual blurring was an atypical presentation, ICIs are known to induce or exacerbate PNS, making it an important differential diagnosis (Wang et al., 2022). While paraneoplastic antibody testing was not performed, the detection of low-titer MOG-Abs in both serum and CSF, together with the close temporal link to ICI administration, supported a diagnosis of MOG-Ab related irAE. This diagnosis was established through multidisciplinary collaboration between neurology and ophthalmology, underscoring the value of a team-based approach in managing complex irAEs. Ophthalmologists should be aware of this entity in oncology patients presenting with rapid vision loss following medication initiation.

Although neurological irAEs associated with ICIs are rare, previous reports have described neuromyelitis optica spectrum disorders (Khimani et al., 2022), MS (Gerdes et al., 2016), and other CNS demyelinating diseases (Pillonel et al., 2019) following ICI treatment. In addition, Marin et al. conducted a review of ICI-induced neurological adverse events, in which CNS demyelinating diseases accounted for 2% (8/428) of cases (Marini et al., 2021). Furthermore, a recent case report described a middle-aged male patient with malignant melanoma who developed lower limb weakness accompanied by dizziness and upper limb numbness 1 week after receiving pembrolizumab, another PD-1 inhibitor. The patient was ultimately diagnosed with MOGAD based on MRI findings and antibody testing (Liu et al., 2022). Although the clinical manifestations differed from those in this case, both reports confirm that ICIs can trigger MOGAD. This case expands the documented range of ICI-induced neurological complications and constitutes a report of a link between tislelizumab and MOG-Ab-associated ON.

Why do we attribute the patient’s condition to tislelizumab rather than carboplatin and etoposide? Carboplatin-induced optic neuropathy, though rare, has been documented in the literature. A recent case report described a patient who developed irreversible bilateral optic neuropathy within 3 days of carboplatin therapy; despite high-dose steroid intervention, visual acuity declined to light perception in one eye and 20/60 in the other, with no improvement at 1-year follow-up. The authors proposed arterial ischemia as the likely underlying mechanism given the irreversible nature of the vision loss (Shihadeh et al., 2024). In our patient, acute bilateral vision loss occurred within 24 h after the first treatment cycle containing carboplatin, etoposide, and tislelizumab (3 September 2025). However, several critical clinical features argue against carboplatin or etoposide as the causative agents. First, after treatment with high-dose steroids and rituximab, the patient’s visual acuity significantly improved and fundoscopic findings (retinal hemorrhages) resolved, and he remained stable without any visual recurrence during subsequent cycles of carboplatin/etoposide chemotherapy (without further tislelizumab). This contrasts sharply with the irreversible, progressive course typical of carboplatin-induced optic neuropathy (Shihadeh et al., 2024). Second, the detection of MOG-Ab strongly supports an immune-mediated demyelinating process characteristic of tislelizumab-related irAE, rather than an ischemic mechanism. Third, etoposide is primarily associated with peripheral neuropathy and very rarely with ocular toxicity; no well-documented cases of etoposide-induced optic neuropathy or MOGAD have been reported. Therefore, the independent or synergistic pathogenic potential of carboplatin and etoposide can be reasonably excluded, and tislelizumab is the most likely causal agent.

The successful treatment of this case confirms the core principles of current guidelines for managing high-grade irAEs and reflects individualized escalation based on disease severity (Shi et al., 2020). The primary and crucial step was the immediate and permanent discontinuation of tislelizumab to completely remove the pathogenic trigger. Second, early and adequate glucocorticoid pulse therapy was administered to rapidly and potently suppress uncontrolled immune inflammation and maximize the preservation of optic nerve function. Finally, the early addition of rituximab, which reduces pathogenic antibody production at the source, constituted a key individualized intensive treatment strategy (Bai et al., 2021). For critically ill patients with double-antibody positivity, as occurred in our case, the early combination of rituximab with steroid pulse therapy formed a “double-hit” immunosuppressive approach, which was likely critical in reversing the life-threatening condition.

Since PNS could not be fully excluded in this patient due to the absence of tumor-related antibody testing, future cases with similar presentations should incorporate comprehensive paraneoplastic antibody screening to enable early diagnosis and appropriate management. As a single-case report, this study has limited generalizability, and the underlying mechanisms, risk factors, and optimal management of PD-1 inhibitor-induced MOGAD require elucidation through larger-scale studies. Consequently, we recommend that any patient developing suspected neurological symptoms following ICI therapy undergo prompt and comprehensive evaluation, including testing for neuroimmune antibodies. This approach, which accounts for individual autoimmune risks, is essential for enhancing the safety and efficacy of immunotherapy.

## 4 Conclusion

This case confirms that following tislelizumab treatment, rare and vision-threatening OirAEs, including MOG-Ab-associated ON, can occur. Our experience demonstrates that successful management depends on heightened clinical vigilance for such rare irAEs, early multidisciplinary team involvement in diagnosis, and the prompt initiation of an intensified immunosuppressive regimen, anchored by permanent ICI discontinuation. In the current era of widespread immunotherapy use, the occurrence of new and rapidly progressive neurological symptoms should prompt prioritization of irAEs in the differential diagnosis. Otherwise, ophthalmologists should be alert that in oncology patients presenting with acute vision loss following ICI administration, differential diagnoses should not be limited to primary ophthalmic disorders; the possibility of OirAEs must also be considered.

## Data Availability

The original contributions presented in the study are included in the article/supplementary material, further inquiries can be directed to the corresponding authors.
